# Detection of Gunshot Residues Using Mass Spectrometry

**DOI:** 10.1155/2014/965403

**Published:** 2014-05-22

**Authors:** Regina Verena Taudte, Alison Beavis, Lucas Blanes, Nerida Cole, Philip Doble, Claude Roux

**Affiliations:** Centre for Forensic Science, University of Technology, P.O. Box 123, Broadway, Sydney, NSW 2007, Australia

## Abstract

In recent years, forensic scientists have become increasingly interested in the detection and interpretation of organic gunshot residues (OGSR) due to the increasing use of lead- and heavy metal-free ammunition. This has also been prompted by the identification of gunshot residue- (GSR-) like particles in environmental and occupational samples. Various techniques have been investigated for their ability to detect OGSR. Mass spectrometry (MS) coupled to a chromatographic system is a powerful tool due to its high selectivity and sensitivity. Further, modern MS instruments can detect and identify a number of explosives and additives which may require different ionization techniques. Finally, MS has been applied to the analysis of both OGSR and inorganic gunshot residue (IGSR), although the “gold standard” for analysis is scanning electron microscopy with energy dispersive X-ray microscopy (SEM-EDX). This review presents an overview of the technical attributes of currently available MS and ionization techniques and their reported applications to GSR analysis.

## 1. Introduction

Scanning electron microscopy (SEM) for the analysis of inorganic gunshot residues (IGSR) was introduced in 1974 by the Aerospace Corporation [1]. SEM coupled with wavelength- or energy-dispersive X-ray detection (WDX or EDX, resp.) has since been extensively applied to GSR analysis [2–8] and has become the internationally accepted analysis method. The use of SEM-EDX analysis is highly advantageous as characteristic elemental composition and morphology of GSR particles can be obtained using this nondestructive method [9]. The reliability of SEM techniques is based on the detection of lead (Pb), antimony (Sb), and barium (Ba) in discrete particles originating from the primer. Over the last 15 years, lead- and heavy metal-free ammunition has been increasingly commercialized to minimise the exposure of frequent shooters to dangerous airborne levels of lead and other toxic metals present in primers and other parts of the ammunition. As a consequence, there is a potential for false negative results when analysing GSR using SEM-EDX [10–13]. Another limitation of SEM-EDX for GSR analysis is that the particles found in IGSR can also be derived from a number of environmental and occupational sources including brake linings [14, 15], fireworks [16–18], paints, and cartridge-operation occupations [19, 20]. These sources have been demonstrated to generate IGSR-like particles and may contribute to the risk of false positives in some situations.

For these reasons, it has become necessary to refocus on the full informational content of GSR as a forensic trace and not only on those compounds that are easily analysed by SEM-EDX. In other words, it may be necessary in some cases to not only analyse IGSR but also analyse organic GSR (OGSR). The information derived by the combined IGSR and OGSR analysis has the potential to overcome the issues related to false positives and false negatives identified above. As a result, significant efforts have been made to improve the detection of OGSR and many of these methods rely upon mass spectrometry (MS).

Mass spectrometry is a highly sensitive and selective analytical technique used to detect and quantify elements and/or compounds and elucidate organic structures [21]. Another advantage of MS is its applicability to using a library database. Such a library database, either purchased or customized, can be used to automatically compare and match the compounds of interest based on their spectra [21].

A mass spectrometer can be divided into three sections: the ion source, where gaseous species desorbed from condensed phases are ionized; the analyzer, where the generated ions are separated according to their mass-to-charge ratio (*m*/*z*); and the detector, where the signal is amplified and transformed into a spectral form. Different ion sources, analyzers, and detectors have been developed providing different sensitivities and resolutions.

Ion sources are commonly divided into soft and hard ionization techniques, where soft ionization techniques are very low-energy transfer processes and hard ionization techniques are relatively high-energy transfer processes. Soft ionization techniques are usually preferred for OGSR analysis as explosive compounds used in propellants are relatively unstable and prone to fragmentation. Examples of these soft ionization techniques include electrospray ionization (ESI), atmospheric pressure chemical ionization (APCI), and desorption electrospray ionization (DESI) [21].

Following ionization, a mass analyzer separates the ions based on their mass-to-charge ratio. Several mass analyzers exist, which are distinguished by the interaction between the ions produced and electric and/or magnetic fields, which leads to their separation.

The final step involves a detector, which is most commonly an electron multiplier. Analyte ions reaching the detector collide with a charged anode generating a cascade of electrons which are released and detected [21].

When selecting an MS, a variety of important factors should be considered such as the *m*/*z* range to scan, sensitivity, resolution, vacuum system, and gas supply. Additional considerations include whether or not a fragmentation pattern is required and analysis costs.

This review provides an overview of the technical attributes and analytical properties of different MS techniques and their reported applications to GSR analysis. A list of analytical techniques which have been applied to GSR analysis and their abbreviations can be found in Table 1.

## 2. Mass Spectrometry Approaches for OGSR

Although all organic compounds found in ammunition can contribute, OGSR mainly originates from propellant powder [22]. Smokeless powders consist predominantly of nitrocellulose (NC) combined with other explosive compounds and additives. These additives include stabilizers, plasticizers, flash inhibitors, coolants, moderants, surface lubricants, and antiwear additives [22]. They are used to increase the stability and workability and to modify the burn rate [13, 23]. Levels of additives present range from trace amounts up to 50% of the power mixture [24]. The molecular structure of these compounds can vary, which is an important consideration when choosing a suitable ionization technique. While explosives are mainly nitrated compounds, many stabilizers contain amine groups and plasticizers are often phthalates. Due to their different chemical properties, different ionization techniques are preferable. Nitro groups are strongly electrophilic and explosives therefore commonly produce a negative ion signal. Most of the additives used in smokeless powders produce intense positive ion signals, for example, the stabilizer diphenylamine (DPA) and its derivatives [25, 26].  Table 2 lists common organic explosives and additives used in the manufacture of smokeless powders and primers.

Sensitivity and selectivity play an extremely important role in the detection of OGSR as organic additives are in low abundance and consequently occur only in very low concentrations in real case samples, typically in the microgram to nanogram range. DPA, for example, is present at around 1% or below in smokeless powder and has been detected at the low microgram to nanogram level in OGSR [22, 28–30]. Similarly, EC, 2-NDPA, N-nDPA, and 4-NDPA have been detected in the low microgram range in OGSR [28], while MC on the hands of shooters has been detected at levels of between around 3 and 19 ng [31]. Since additives are used at different concentrations during manufacture, the quantitative analysis of smokeless powder and comparison to GSR can lead to discrimination between smokeless powders. Further, it may assist in the identification of the ammunition type used [32] and the estimation of the time since discharge [33].

Compounds of interest in OGSR are usually present in complex sample matrices. As a result, separation is typically required before detection. Most MS techniques are coupled with chromatographic systems such as GC or LC. The high mass resolution of some spectrometers may avoid the necessity for full baseline separation unless isomers are present [34].

The following section discusses reported MS ionization techniques for OGSR analysis.

### 2.1. Electron Impact (EI)

In EI ionization, electrons are emitted from a hot filament and accelerated through 70 eV before interaction with the molecule of interest. Since the energy required for ionization is much lower than the kinetic energy of the electron, EI generally leads to a strong fragmentation pattern and the molecular ion may have a low abundance or may not be present at all. Therefore, this method is considered a hard ionization technique. The ions produced are repelled and focused by plates with defined potentials before entering the analyzer [21].

Volatile compounds in GSR can be used for the investigation of time since discharge. For this purpose Weyermann et al. detected 32 volatile compounds in a 9 mm spent cartridge by SPME-GC coupled to a Q-MS [33]. Q-MS is one of the most commonly used analytical techniques due to its relatively low cost [21]. The detector most commonly used with Q-MS is the high-energy dynode in which all ions reaching the detector produce a similar electrical response and no mass discrimination occurs (ions with different *m*/*z* produce different signals) [21]. GC was used by Weyermann et al. as it allows direct introduction of the SPME fiber to the injection port, making it suitable for analysis of volatile compounds. Six target compounds were chosen and tested for feasibility as determinants of the time since discharge in a 32-hour time frame. The results were promising; however the poor reproducibility due to the use of SPME was a significant drawback, particularly for small quantities [33]. Typically the use of a hard ionization technique such as EI is not suitable for OGSR; however in this study only propellant additives were analysed, not the thermally labile explosives present in OGSR.

To improve the reproducibility of measurements and recovery of OGSR by SPME, Dalby and Birkett examined different SPME fiber types using GC-Q-MS [35]. The developed method was able to identify 27 compounds using the National Institute of Standards and Technology (NIST) database. Of the seven different SPME fibres examined, polydimethylsiloxane/divinylbenzene (PDMS/DVB) was found to be the most suitable based on the extraction of DPA, 4-NDPA, EC, NG, and DBP [35].

Headspace analysis of volatile and semivolatile additives in smokeless powders using SPME-GC coupled to an ion-trap MS and IMS was studied by Joshi et al. [36]. The objective was identifying target compounds for testing the feasibility of generating headspace profiles to facilitate differentiation of smokeless powders. Most ion-trap mass spectrometers are QITs also called Paul ion-traps. These instruments offer high sensitivity (1–10 pg mass detection limit) and are generally 10 to 100 times more sensitive than Q-MS. Ions are deflected on three-dimensional orbits by a ring electrode and two end-cap electrodes and then expelled based on their *m*/*z* ratio and the applied radio frequency. As for Q-MS, the detector commonly used with QIT-MS is a high-energy dynode [21]. A drawback of QIT-MS is that the dynamic range of ion intensities is limited [37], and only ~5% of the injected ions which are trapped as cavities can only hold a limited number of ions before the allowed space charge is exceeded. However, the low detection limits and the possibility to conduct tandem MS experiments make it an attractive approach. Sixty-five single- and double-base powders were examined and their headspace compounds were qualified and quantified with the use of a NIST database. The stabilizer DPA was present in most of the powders, accompanied by small amounts of its derivatives. The stabiliser EC was present in almost half of the powders tested, while MC could only be detected in 8% of samples tested. In double-base powders, the explosive NG was the major peak. The reproducibility of the SPME-GC-MS method was evaluated and shown to facilitate differentiation of smokeless powders based on the quantification of OGSR. When comparing the performance of SPME-IMS with SPME-GC-MS, it was noted that using IMS detection limits were higher and the potential for false-negatives was increased. Some compounds could not be detected, similar drift times of compounds preventing full identification or quantification. Drawbacks of the SPME-GC-MS method are that NG could not be detected due to its thermal degradation under the high temperatures in the GC oven and the relatively long analysis time in comparison to IMS. However, this method is promising for the analysis of headspace compounds of smokeless powders [36].

Studies by Dalby and Birkett [35] and Joshi et al. [36] have also demonstrated the benefits of database matching for identifying compounds present in smokeless powders.

Although EI was shown as useful for the detection of volatiles and semivolatiles present in OGSR coupled to a GC-MS, more commonly soft ionization techniques are applied. For compounds prone to fragmentation, such as high explosives, EI can prevent positive identification and is therefore not the method of choice.

### 2.2. Chemical Ionization (CI)

One of the earlier developed soft ionization techniques is CI. For CI, the ionization source is filled with a reagent gas such as methane, isobutane, or ammonia at a pressure of around 100 Pa. Energetic electrons convert the reagent gas into a variety of different reactive ions. These ions then interact with the molecules of interest, which leads to the formation of ionic species. CI is a softer ionization technique than EI and the molecular ion is commonly identified [21].

In two early studies of the application of MS to OGSR analysis, MSF-MS was coupled to a gas chromatograph to detect different OGSR compounds, that is, NG, 2,4-DNT, DPA, EC, and DBP, in unburnt and burnt smokeless powders and acetone hand swabs after shootings with different ammunitions. In an MSF-MS accelerated ions are deflected towards a detector based on the interaction with a magnetic field that is perpendicular to the direction of their way of travel. The analyzer tube is maintained under vacuum (~10^−5^ Pa) in order to reduce collisions between the ions of interest and gas molecules. Only ions with a specific *m*/*z* ratio reach the detector, while heavier or lighter ones are deflected too much. A range of different *m*/*z* ratios can be scanned by varying the magnetic field strength. A common detector in MSF-MS instruments is the electron multiplier detector. The incoming ions cause a cascade of electrons that are further multiplied by a series of dynodes. The electrons reach the anode where the current is measured [21]. A comparison of CI with methane and CI with helium against EI was performed by Mach et al. [23]. CI with methane proved to be the softest ionization technique in that it produced protonated molecular ions for all tested compounds while with helium, both EI and CI gave only fragment ions for NG and DBP. It was possible to identify the presence of smokeless powder compounds; however, type and cartridge calibre could not be assigned [23]. Using the optimized ionization technique, the second study investigated burnt flakes of smokeless powders and acetone hand swabs. Burnt flakes could be identified as originating from smokeless powders; however, the composition in flakes and original powder varied. These changes may result from degradation during shooting or contamination in the firearm. Swabs moistened with acetone proved to be unsuitable for the collection of DPA from hands due to a very low recovery efficiency when low concentrations were present [38].

Gas chromatography as applied by Mach et al. [23, 38] is however not the method of choice for the analysis of OGSR as the high temperatures applied in the GC oven can lead to thermal degradation of instable organic explosives. Alternate approaches using LC-MSF-MS have also been developed [39]. After comparison of CI in negative and positive modes, the negative mode was identified as having superior sensitivity for the explosives tested by Parker et al. Lower temperatures were reported to reduce fragmentation of RDX, TNT, tetryl, and PETN [39].

Positive CI coupled to an MSF-MS was applied to the analysis of explosives commonly encountered in OGSR including NG, DEGN, TNT, tetryl, 2,4-DNT, RDX, PETN, and ammonium nitrate by Yinon and Hwang [40]. Different mobile phases evaluated showed no significant differences. The protonated molecular ion was detected for all compounds, except tetryl. Adduct ions formed with methanol or acetonitrile (depending on the mobile phase used) and fragmentation ions were further characterized. A drawback of the method was the reduced sensitivity (2 orders of magnitude) due to the use of direct liquid injection with only 1% of the LC eluent entering the ion source. Limits of detection were between 1 and 10 *μ*g injected (10–100 ng in the ion source) [40].

### 2.3. Electrospray Ionization (ESI)

Alternative soft ionization techniques including ESI have been developed which can be used at atmospheric pressure. During ESI, an electrical field (ca. 3–5 kV) is applied to a liquid, which passes through a stainless steel capillary. The resulting charged small droplets are ionized by the formation of adducts with added salts, usually ammonium salts. After solvent evaporation, the ions are introduced into the mass spectrometer [37]. LC and CE may be coupled to this ionization technique as it occurs under atmospheric pressure.

ESI coupled to QIT-MS was applied to the characterization of smokeless powders by quantitative analysis of several additives, including DPA, some of its derivatives, DBP, and DEP in order to aid in the determination of which ammunition was used [41]. Mathis and McCord used LC for the separation of the smokeless powder with ammonium acetate added to the mobile phase to enhance ionization in positive ESI mode. It was found that DBP suffered from “in-source” fragmentation, which occurs when ions are transferred from the atmospheric pressure source to the MS. Following optimization, eleven unburnt smokeless powders could be characterized based on the presence or absence of certain additives or if the composition was similar on the differing concentrations of specific compounds. This method was unable to detect DMP and MC in any powder sample tested [41].

The influence of ammonium salts on the formation of anionic adducts was evaluated in another study by Mathis and McCord [42] who found that both specificity and selectivity could be enhanced for the high explosives tested by using specific ammonium salts. It was found that EGDN, NG, PETN, and RDX built the most stable adducts with ammonium nitrate, while HMX had the most abundant ion (*m*/*z* 331) with ammonium chloride. Superior selectivity was obtained when the ammonium salt was added to the mobile phase precolumn compared to addition postcolumn. Sensitivity enhancement was independent of the ammonium salt concentration. This method showed potential for the detection of high explosives in complicated sample matrices without extensive sample preparation as a result of the improved sensitivity and selectivity based on adduct formation [42].

Liquid chromatography coupled to Q-MS over an ESI interface has been used to examine the recovery of different explosive residues (present in OGSR) from cotton swabs by Thompson et al. [43]. The authors used LC-Q-MS (RDX, HMX, tetryl, NG, TNT, DNT, and PETN) and GC-MS (EGDN) to confirm the identity of explosives previously screened by LC with ultraviolet (UV) detection. For LC-Q-MS, ESI in negative mode was used and ammonium nitrate was added in order to enhance sensitivity and selectivity as described above [42]. However, the instrument response was nonlinear over the measurement range and only a two-point calibration was performed. Consequently, LC-Q-MS proved unsuitable for quantifying the explosives and quantitative measurements were achieved using LC with UV detection. The results showed that water extraction followed by SPE for preconcentration reduced interference, providing better selectivity [43].

Several studies were conducted focusing on ESI coupled to tandem MS for the detection of OGSR. Tandem mass spectrometry, a relatively recent introduction, can serve as a more precise identification tool as parent and daughter ions are analysed and higher selectivity is obtained. The high selectivity offered by MS/MS can allow the omission of a preceding chromatographic system when isomers are not present. The superior sensitivity and selectivity offered by MS/MS compared to MS have resulted in its increasing application to GSR analysis [44, 45].

Wu et al. developed a tandem QqQ technique for the detection of MC, a stabilizer that is usually found in Chinese ammunition [29, 31]. The stabilizers MC and EC are considered to be the most characteristic compounds for OGSR [23, 28] and their detection is therefore of high evidential value. QqQ-MS instruments consist of a series of three connected quadrupoles. In MS/MS, the first quadrupole filters the ions for the chosen precursor ion with a known *m*/*z* ratio. The second quadrupole has only radio frequency applied to it and functions as a collision cell, and the third quadrupole serves as a second mass filter and can scan or filter the fragments produced. Ions unrelated to the target compounds are filtered out. After ESI (+), the two ions of interest were the precursor ion [M+H]^+^ with *m*/*z* 241 and the product ion [M-(N(C_6_H_5_)(CH_3_))^−^]^+^, *m*/*z* 134, detected in multiple reaction monitoring mode (MRM). These precursor and fragment ions were selected as the abundance of the product ion should be maximal and interference from the actual sample was avoided. The latter was confirmed as no samples taken from the hands of volunteer shooters showed any interference with MC using these two ions. Optimising the QqQ-MS conditions, collision gas flow rate, and the focusing lens, a detection limit of 60 pg injected for MC was obtained with excellent linearity (*R* ^2^ > 0.999) and reproducibility. The method was sufficiently sensitive and selective to detect MC in all hand swabs of shooters even after 100-fold dilution, eight hours after shooting, or when hands were washed under running water (no soap) before collection and enabled the direct analysis of the extracts in less than five minutes. However, strong absorption of MC onto the injection tube and the instrument increased the detection limit [29].

Wu et al. extended the above tandem QqQ-MS technique to analyse NG [31]. Three ions were monitored for NG in negative mode as follows: the precursor ion [M−H]^−^ at *m*/*z*/226 and the two product ions [M−HNO_3_]^−^ and [NO_3_]^−^ at *m*/*z* 163 and 62, respectively. For MC, the same two ions described above with three additional fragmentation ions at *m*/*z* 106, 77, and 51 were used as characteristic ions. Eight samples each from hands of shooters and nonshooters were analysed; the two groups were easily distinguished by this method. Detection of MC concentration was time-sensitive; after two hours only 25% of the initial concentration of MC could be detected; however, only minimal changes were observed from two to eight hours [31].

Tong et al. extended the QqQ-MS/MS method to the detection of DPA, 4-nDPA, 4-NDPA, N-nDPA, and 2,4-DNDPA. DPA in combination with its nitrated and nitroso derivatives is considered unique to smokeless powders and therefore of high evidential value, but not when found alone. These nitrated and nitroso species arise from the decomposition of nitrocellulose and nitroglycerine in smokeless powders [45]. Here, a flow injection system directly infused the samples into the MS. Using positive ESI mode, two ions were selected (a precursor, i.e., the molecular ion, and the most abundant fragment ion) for each of the five compounds in MRM scanning mode. Different smokeless powders and spiked hand samples were analysed and the amounts of DPA and its derivatives were quantified. It was shown that 4-nDPA and 2,4-DNDPA could not be detected in any smokeless powder samples. The relative high reactivity and instability of 4-nDPA and the rare formation of 2,4-DNDPA were assumed to account for these results. Limits of detection were between 0.5 and 2.5 ng/mL [45]. Although this method showed great potential for the detection of DPA and two of its derivatives in GSR samples, only spiked hand samples were tested, but analysis of real case samples has yet to be reported.

The studies by Wu et al. and Tong et al. demonstrate the great advantage offered by MS/MS enabling the possibility of omitting a preceding chromatography step. This is possible since the selectivity is greatly enhanced and interference can mostly be eliminated [29, 31, 45].

Another study focusing on DPA and its derivatives as well as AK II, EC, and MC using a new sample preparation with SPE was conducted by Laza et al. [28]. Swabs moistened with isopropyl alcohol/water were used to sample test-shooters. These swabs were extracted and preconcentrated using SPE and the extracts were injected onto an HPLC, which was coupled to QqQ-MS/MS by an ESI interface. In MRM mode, the two tested coeluting DPA derivatives (4-NDPA, N-nDPA) were well distinguished. Samples taken from shooters and unfired propellant powders were analysed showing promising results for real case analysis; however neither MC nor AK II could be detected in any samples [28].

All previous studies proved the utility of ESI-QqQ only for the detection of additives present in OGSR. Perret et al. extended this and developed an LC-QqQ tandem MS method for the combined analysis of explosives and stabilizers in OGSR [44]. These include RDX, NG, TNT, PETN, DPA, and EC. After LC, ESI was used in either negative mode (NG, RDX, PETN, and TNT) or positive mode (DPA, EC) to produce the ions required for MS/MS. Using ammonium acetate as the buffer, ionization was more stable, with a more reproducible signal, lower background, and an improved S/N ratio. Collision energy and declustering energy, which can help reduce “up-front” collision-induced dissociation (CID), were optimized for the production of precursor and fragment ions. For NG, RDX, and PETN, the base peak was the acetate adduct. The base peak for TNT was the deprotonated molecular ion and for DPA and EC the protonated molecular ions. This method was applied to the extraction of explosives from hand swabs. Methanol gave superior recovery and efficiency of extraction when used for retrieval of explosives from either cotton or alcohol swabs. Explosives were detectable six hours following handling [44].

The previous studies demonstrate that the use of ammonium salts can be beneficial for OGSR detection as it can improve sensitivity and selectivity and the formation of adducts can make extensive sample preparation redundant [41, 43, 44].

ESI is applicable to a wide range of compounds present in OSGR and has been extensively applied with single and tandem MS. However, particularly when compared to APCI, this technique has some drawbacks such as limited ionization of nitroaromatics and generally lower response for nitrocontaining explosives [44, 46, 47]. Since APCI enables the ionization of most compounds likely to be present in OGSR, it appears to provide superior results for OGSR analysis than ESI in most circumstances [46, 48].

### 2.4. Atmospheric Pressure Chemical Ionization (APCI)

Atmospheric pressure chemical ionisation like ESI is a soft ionization technique that can be applied under atmospheric pressure and therefore can be easily coupled with LC or CE instruments. The interface is very similar to ESI, except that vaporization takes place under heating instead of applied voltage.

The differences between ESI and APCI coupled to a QIT-MS for the combined detection of nitramines, nitrate esters, and nitroaromatics, that is, NG, PETN, RDX, HMX, tetryl, TNT, 2,4-DNT, TNB, and 1,3-DNB, were studied by Tachon et al. [47]. An LC separation method was used followed by negative mode ionization with the view to compare ESI with APCI. Poor results were obtained for the ionization of nitroaromatics in ESI. This is consistent with the relatively high limit of detection (LOD) found for TNT using ESI-QqQ [44]. Adduct formation with formate was predominantly observed for nitrate esters and nitramines with both ESI and APCI. The thermally labile nitrate esters tend to degrade under the heated vaporization applied in APCI. Consequently, the intensities of nitrate esters were found to be larger in ESI than APCI. However, for the combined analysis of all three groups of nitroexplosives, APCI proved to be superior to ESI. The developed method showed LODs between 0.04 and 1.06 ng/*μ*L (with 10 *μ*L injected). However, when simulated real case samples obtained from motor oil extracts were studied, only qualitative results could be obtained due to matrix effects [47].

Another study using LC separation and negative mode APCI coupled to a QIT-MS for the detection of 21 explosives covering nitroaromatics, nitramines, and nitrate esters was conducted by Holmgren et al. [49]. Chlorine adducts of the nonaromatic nitrates were formed through the addition of dichloromethane to the mobile phase. Using the optimized method, LODs were obtained between 0.5 ng and 41.2 ng (PETN) injected [49].

The mass accuracy of ToF instruments can enable the relatively confident identification of a great number of compounds, including isomers. In ToF, ions are separated on the basis of the influence of their mass-to-charge on their relative velocities. Kinghorn and Milner tested the suitability of ToF-MS for a wide range of explosives some of which can be found in OSGR. ESI and APCI in both positive and negative modes were evaluated for nitroaromatics, nitramines, nitrate esters, and peroxides. As identified by Tachon et al. also using QIT-MS [47], neither ESI nor APCI is universally applicable to the ionization of all compounds. The study by Kinghorn and Milner supported this outcome; however, APCI in negative mode showed the best results for all tested compounds with the exception of HMTD, 3,3-dimethyl-1,2-dioxacyclopropane (TATP), and EC which were not detected. This method showed high mass accuracy enabling the identification of HMX in APCI (+) and the reduction of background noise and therefore LODs when compared to Q-MS [46].

The feasibility of using LC-tandem QqQ-MS for the quantitative analysis of OGSR was also studied by Yang et al. [50]. An LC-QqQ-MS method was developed and validated for the qualitative and quantitative detection of three representative compounds: NG, DNT, and EC. NG and DNT were ionized in APCI (−) and EC in ESI (+). The method showed excellent linearity and low LODs (0.01–0.5 pg) and was further tested on samples from fired cases, gun powders, and hand swabs from shooters. In fired cases and gun powders samples, all three compounds could be detected. DNT is not often used in gun powder and was detected at a level three times below the limit of quantification (LOQ) precluding quantitation. In hand swabs, only EC and NG were detected, with the nonoptimized swabbing technique blamed [50].

DeTata et al. applied tandem QToF-MS coupled with LC for the identification of over 50 organic explosives and propellants [34]. In comparison to QqQ systems which can be run in four different modes, the QToF-MS is commonly run either in precursor (MS) or product ion mode (MS/MS). One of the main advantages of QToF-MS over QqQ-MS is the higher mass resolution and specificity and a resulting higher S/N ratio [51]. However, the best absolute sensitivity to date is still provided by QqQ-MS [51]. APCI was used in positive and negative modes since the majority of nitrocontaining explosives give a greater response with APCI (−) than ESI as shown by previous studies [46, 47]. All 64 explosive standards were detected (as formate adducts), including many OGSR compounds. The limits of detection for all compounds tested were between 0.001 ng (HMX) and 1000 ng (nitrourea and triethylene glycol dinitrate), which is comparable or lower than that obtained by QqQ-MS or ToF-MS analysis in previous studies [46, 49]. Analyte data including accurate mass, isotope ratios, and retention time were fed into a custom personal compound database and library (PCDL). Using this library, samples could be rapidly screened followed by further identification using the MS/MS precursor and product ion data [34]. APCI gave superior performance for analysis of OGSR in comparison to other ionization techniques. Only nitrate esters showed lower intensities than in ESI mode due to their decomposition under the higher temperatures applied [47].

### 2.5. Atmospheric Pressure Photoionization (APPI)

As discussed above, APCI has been successfully applied to the ionization of nitrate esters, nitramines, and nitroaromatics. Another ionization technique with promising features for analysis of organic explosives is APPI [52]. The structure of APPI is similar to that of APCI; however, ionization is induced by photons at 10.0 or 10.6 eV emitted from a krypton lamp [53]. These energy levels have the advantage of greatly reducing the ionization of air and common HPLC solvents. Moreover, the direct photoionization of analytes in APPI can enable the ionization of compounds which have proven refractive to other sources such as ESI or APCI [53]. Crescenzi et al. compared APCI and APPI in negative and positive modes for the potential identification and quantification of a range of organic explosives including those present in OGSR [53]. In APCI more basic compounds such as diamino compounds are ionized in positive mode as they produce no signal in negative mode [46]. In APPI, some of these compounds can be analysed in negative mode. This however does necessarily represent an advantage as their detection limits are generally higher in negative mode. For example, 2,6-diamino-4-nitrotoluene (2,6-DA-4-NT) could not be detected using APCI (−) but showed when APPI (−) was used. However, the detection limit in APPI (−) was 74 ng injected in comparison to 1.6 ng injected when using APPI (+) or 2.7 ng injected when using APCI (+) [53]. For compounds that could be analysed with both interfaces, LODs were found to be lower overall with APCI. A major drawback for APPI was that no cyclic nitramines and nitrate esters could be detected. Therefore, APCI was selected for further optimization. LODs using APCI were between 2.5 pg/mL (2,4-DNT, 2,6-diamino-4-nitrotoluene; 2,6-DA-4-NT) and 563 pg/mL (PETN). A great advantage of the developed method is that no sample pretreatment is required due to an online extraction method based on a six-valve system allowing a sample volume up to 100 mL [53]. Conclusively, APPI is unlikely to find wide applicability to OGSR analysis as cyclic nitramines and nitrate esters, which are present in OGSR, are not detected.

### 2.6. Laser Electrospray Ionization (LEI)

In an attempt to overcome limitations observed with alternate methods, LEI coupled with a ToF-MS was evaluated for the characterization of smokeless powders [54]. The smokeless powders were evenly deposited on a double-sided adhesive and raster scanned with femtosecond laser shots. Protonated molecular ions were quantified in positive mode, of which [EC+H]^+^ was found to be the main distinguishing component. Principal component analysis (PCA), a multivariate data reduction technique, classified all five tested powders using only three randomly selected mass spectra as a training set. A great advantage of coupling ESI with laser vaporization was that no sample preparation of the smokeless powders was required [54].

### 2.7. Secondary Ion Mass Spectrometry (SIMS)

Mahoney et al. also utilised PCA when examining the application of ToF secondary ion mass spectrometry (ToF-SIMS) for the differentiation of three different smokeless powders and six black powders based on full scan positive ion ToF-SIMS spectra [55]. Compounds including EC, DNT, NG, and DBP were identified by library matching using The Static SIMS library as well as comparison to standards. Peaks corresponding to unidentified compounds could also be used for powder characterization. The spatial distributions of some compounds present in the tested powder grains were determined using SIMS. It should be noted that this tool does not seem necessary for the characterization of smokeless powders since varieties in the manufacturing process may have an influence on the distribution of compounds [55].

### 2.8. Desorption Electrospray Ionization (DESI)

In 2004, a new ionization technique, called DESI, was introduced and has since been applied to the analysis of a wide range of organic compounds including some present in GSR [56–59]. This ionization technique enables the* in-situ* analysis of various surfaces under ambient conditions requiring minimal sample pretreatment [60, 61]. When coupled with MS, DESI has been of great interest to GSR analysis since it allows the direct analysis of OGSR from different surfaces such as GSR stubs, skin, or clothes [58]. It may therefore be possible to combine IGSR analysis by SEM-EDX with OGSR analysis from the same stub.

Zhao et al. coupled a QIT-MS instrument in tandem MS mode to a DESI source for the detection of MC and EC from different surfaces including hands, hair, glass, rubber gloves, leather gloves, towel, medical gauze, and adsorbent cotton [30]. DESI was operated in positive mode to produce the protonated parent ions. It was possible to produce the parent ion from all different surfaces tested. The S/N values of DESI-MS and DESI-MS/MS were compared for MC and proved that tandem MS greatly lowers the detection limits, that is, approximately eight times. Using tandem MS, LODs were found to be between 5–60 and 6–70 pg/cm^3^ for MC and EC, respectively. The developed method was able to distinguish between 10 shooters and 10 nonshooters by the analysis of their hands for MC and EC. Spiking skin with possible interfering substances including milk, soft drinks, and dust showed that the method was sufficiently sensitive to detect MC. MC could still be detected from hands after 12 hours and after six hand washings under running water [30].

The ambient ionization technique of DESI coupled to QToF was evaluated for DPA, EC, and MC by Morelato et al. [58]. A range of unfired smokeless powders, as well as GSR stubs after shootings, were analysed. DPA, nitrated DPA, and EC could be detected in the tested powders, however MC could not. On stubs taken after test-firings, only EC was detected. The absence of other compounds was explained by the adhesive surface properties of GSR stubs which might lower the sensitivity. This method showed potential for the combined analysis of IGSR and OGSR but requires further optimization [58].

It is interesting to note that Zhao et al. [30] could detect MC and EC from all tested surfaces, while Morelato et al. [58] experienced difficulties detecting MC from GSR stubs after shooting. This discrepancy is likely explained by the prevalence of MC in Chinese ammunition. In addition, Zhao et al. used a homemade source while Morelato et al. used a commercial available source. The application of tandem MS (i.e., leading to increased sensitivity) [30] could also explain why Zhao et al. detected MC while Morelato et al. [58] did not.

### 2.9. Dual Ionization (ESI and APCI)

Recently, Thomas et al. developed a UHPLC method coupled to a QqQ-MS which enabled the detection of 20 compounds present in OGSR [25]. A dual ionization mode called ESCi (Waters Corporation, Milford, MA) was used which enables the fast switching between ESI and APCI in the same run. Combined with polarity switching, a wide range of compounds can be detected under optimal ionization conditions. Ammonium salt adducts were demonstrated to improve ionization as also previously described [25, 41, 43, 44]. The developed method was applied to the characterization of unburned smokeless powders extracted in dichloromethane for six hours. Powder extracts were analysed and the compounds present were quantified. All tested powders could be distinguished based on their powder profile [25].

A summary of different MS based analytical techniques applied to OGSR analysis can be seen in Table 3. The trend towards atmospheric pressure ionization techniques, that is, ESI and APCI, is reflected in the increasing number of applications of these techniques to OSGR analysis. Additionally, using these techniques in polarity switching mode allowed the highest number of OGSR components to be detected. Quadrupole instruments showed inferior LODs when compared with ion-traps or ToF instruments. However, their cost effectiveness and robustness are advantageous. The highest sensitivity, that is, lowest LODs, was obtained when MS/MS was used with a QqQ mass analyzer [25, 29, 44, 45].

## 3. Mass Spectrometry Approaches for IGSR

The identification of IGSR is commonly achieved by the detection of Pb, Ba, and Sb in discrete GSR particles using SEM-EDX since particles containing these three elements are considered as characteristic for GSR. Other commonly found metals in lead- and heavy-metal containing ammunition are silicon, calcium (Ca), aluminium (Al), cupper (Cu), iron (Fe), sulfur, phosphorus (rare), zinc (Zn), nickel, potassium (K), and tin. However, particles containing these metals in combination with only one or two of the metals characteristic for GSR, that is, Pb, Ba, or Sb, are not considered characteristic of GSR as their presence may be nonfirearm related. Only whole particle populations can provide a better understanding of the origin of a particle [62]. The features of SEM-EDX permit the nondestructive analysis of GSR samples and can provide information about the morphology and chemical composition of a single particle. However, the recent introduction of lead- and heavy metal-free ammunition to the market could limit the applicability of SEM-EDX [10–12, 15, 30, 63, 64]. Metals which can be found in lead-free ammunition include strontium [12], titanium, or Zn [10]. The detection of these metals can be challenging since particles containing metals with a low atomic number, for example, Zn, may remain undetected by SEM-EDX with automated GSR software. Additionally, the detected particles are not characteristic to GSR but only indicative. As a result, different research groups have aimed to detect IGSR by other analytical techniques, some of which are MS based.

This part of the review is divided into sections discussing the applicability of different mass analyzers to IGSR detection.

### 3.1. Time of Flight Secondary Ion Mass Spectrometry (ToF-SIMS)

Offering low limits of detection and high surface sensitivity, ToF-SIMS was examined for its feasibility as a complementary technique to SEM-EDX by Coumbaros et al. [65]. In some cases involving GSR, for example, shootings with 0.22 calibre, there is a lack of three-component particles. Only two- or one-component particles may be present which results in the analysis being inconclusive as to the origin of the particle. In comparison to SEM-EDX, ToF-SIMS offers lower detection limits and the detection of a range of elements that cannot be analysed by SEM-EDX. However, these elements are not necessarily characteristic for GSR and may only be of interest when the origin of a particle is questioned. Using ToF-SIMS, indicative compounds such as Ba in an oxidised form, for example, BaOH^+^, could be detected, offering additional information about the elemental composition of GSR particles. The sensitivity for Ba, K, and Ca was greater and the possibility to etch the particle surface allowed further information in regard to elemental distribution. A disadvantage of ToF-SIMS in comparison to SEM-EDX is the lack of high resolution imaging capabilities. Consequently, ToF-SIMS appeared as a potential technique for complementary analysis of IGSR only when just two- or one-component particles can be detected by SEM-EDX [65].

### 3.2. Inductively Coupled Plasma-Mass Spectrometry (ICP-MS)

Inductively coupled plasma-mass spectrometry was commercially introduced in 1983 and since then has been applied in different fields including forensic chemistry [66]. In the torch of an ICP, a plasma gas (commonly argon) is ionized by an electrical coil. The produced free electrons and Ar^+^ ions are accelerated by a radio-frequency coil and collide with analyte atoms. This collision results in a transfer of energy to the entire gas and the resulting plasma is directed through an interface into a mass spectrometer [21]. The bulk of ICP-MS instruments are equipped with a Q-MS and offer high sensitivity, low detection limits (0.00001–0.0001 ng/g), wide linear dynamic ranges, and relative freedom from matrix interference [67]. For GSR analysis, the focus lies on the detection of Ba, Sb, and Pb. The spectrum of Sb has two isotopes with the masses 121 and 123. The only possible interference is represented by the minor isotope (0.87%) of tellurium and can be easily corrected. The two major isotopes (out of seven) measured for Ba are 137 (11.32%) and 138 (71.66%). Possible interferences for ^138^Ba are the minor isotopes of lanthanum (0.089%) and cerium (0.250%). However, these isotopes have not been measured in GSR samples and can thus be neglected. Lead has four isotopes at masses 204, 206, 207, and 208. The lead isotope ratios cannot be predicted and may be indicative of the origin of the lead. In order to reduce errors resulting from the differences in isotopic distribution, the intensities of the three isotopes at 206, 207, and 208 are commonly added together [68]. The ability to detect a wide range of elements found in GSR and rapid analysis times indicate that this is a promising technique for GSR analysis [67, 68]. Therefore, different research groups have been actively evaluating the feasibility of ICP-MS in IGSR analysis.

#### 3.2.1. ICP-Q-MS

Koons investigated ICP-MS performance for the analysis of GSR from collection swabs [67]. Swabs were spiked with Sb, Ba, and Pb as IGSR and indium (In) and bismuth (Bi) as internal standards to facilitate quantitative analysis. The isotopes of interest were ^121^Sb, ^138^Ba, and ^206+207+208^Pb. The developed ICP-MS method offered excellent linearity for all three elements. Cotton swabs (two swabs per sample) were extracted with 10% (v/v) HNO_3_ solution with heating. This was followed by mixing and centrifugation which showed no uncorrected matrix effects from swab constituents. Consequently, this method gave nearly quantitative recovery results for all three IGSR elements. The use of internal standards reduced signal enhancement due to instrumental drift and signal suppression due to easily ionisable elements, in this case sodium (Na). This is important as Na can be present in GSR samples due to its presence in bleached swabs and as it is a major constituent of human perspiration. The developed method exhibited excellent precision and good accuracy and was able to detect Sb, Ba, and Pb down to 0.5 ng, 0.2 ng, and 1.4 ng, respectively, per swab pair using a 10 mL extraction volume (typically observed levels in GSR are 40–500 ng [69]). These results demonstrated the superiority of ICP-MS compared with graphite furnace atomic absorption spectroscopy (GFAAS) and ICP coupled with atomic emission spectroscopy (AES), both of which have higher LODs. Analysis of GSR swabs taken at a firing range proved the applicability of ICP-MS to IGSR analysis obtaining comparable results to GFAAS and ICP-AES.

These findings were confirmed in another study by Bakowska et al. [70]. The authors used the same extraction procedure and focused on the same isotopes as Koons [67]. Four samples (two swabs each) were taken from the left hand palm, the bottom of the left hand, right hand palm, and the bottom of the right hand of a shooter who had fired a 9 mm semiautomatic gun with 9 mm ammunition. These samples were analysed and the amounts of ^121^Sb, ^138^Ba, and ^208^Pb were quantified. All swab samples showed concentrations of the three IGSR in the *μ*g range allowing the differentiation between samples taken from nonshooter sand shooters. The detection of other elements present in GSR samples was thought to be of value for profiling GSR; however this was not further studied [70].

The analysis of gunshot residue taken from an AK-47 or another type of rifle by ICP-MS was studied by Wang [68]. The author automatically added an internal standard mixture containing lithium (^6^Li), scandium (^45^Sc), ^115^In, yttrium (^89^Y), terbium (^159^Tb), and ^209^Bi through a “Y-piece” to the nebulizer. A similar extraction procedure as reported by Koons [67] was used. The five-point calibration performed showed great linearity and little background noise, typically below 10 ng/L and 20 ng/L for lead. The concentration of the three IGSR in GSR collection swabs was between 0.002 *μ*g/swab (Sb from AK-47) and 5.6 *μ*g/swab (Pb from AK-47) [68].

The analysis of gunshot wounds has also been achieved using ICP-MS [69, 71]. Gunshot residue analysis on deceased bodies can be quite challenging. The identification of GSR can be increasingly difficult when encountering postmortem factors such as decomposition, burial, and insect activity [72]. In this case, the analysis by standard techniques such as SEM-EDX is limited as the oily decomposing tissue surface greatly restricts the uptake of GSR by the adhesive tapes which are commonly used as collection devices for SEM-EDX. Using ICP-MS Lagoo et al. were able to detect GSR in digested tissue at any stage of decomposition during summer and winter, while SEM-EDX has only been able to detect GSR within 24 hours [71]. Udey et al. extended this study and examined the possibility of differentiating bullet types in fresh and decomposed tissues using ICP-MS [69]. After microwave digestion of the tissues, In (for elements below atomic mass 155) and Bi (for elements above atomic mass 155) were added as internal standards. The Q-MS was operated in full scan mode except for the decomposed tissue sample, which was analysed in selected ion monitoring (SIM) in order to increase the sensitivity. Elements such as Cu, Fe, Sb, Ba, and Pb were found to be present in significantly different concentrations in wounds shot with different bullet types and could be used to differentiate control samples from samples taken from gunshot wounds. For fresh tissues, Sb and Pb concentrations were significantly higher in samples from nonjacketed GSR wounds, while Cu was more concentrated in samples taken from tissues shot with jacketed bullets. In decomposed tissues, Cu could again be found in significantly higher concentrations in tissue shot with jacketed bullets, while Pb was more concentrated in wounds from nonjacketed bullets. Sb showed no significant difference in concentrations between wounds from jacketed and nonjacketed bullets for decomposed tissues [69].

Santos et al. used ICP-MS to investigate the relationship between the radial pattern of the GSR deposit around the bullet entrance hole (quantity of Sb, Ba, and Pb) and the firing distance [73]. Using a semiautomatic pistol, five test shots were fired on a cotton tissue with muzzle to target distances between 20 and 200 cm. Samples were then taken by cutting quadrants at four different radial positions and the tissue was digested using HNO_3_. The authors proposed a mathematical model to describe the relationship between the concentration of Sb, Ba, and Pb and shooting distance between 20 and 80 cm. The results suggested that a quadrant cut from the cotton tissue in 4 cm radial position from the entrance hole gave the best results for the estimation of the shooting distance. The concentration of only one element could also be used for estimation of shooting distance [73].

Steffen et al. used ICP-Q-MS to determine if primers could be differentiated by quantitative differences in their Pb-isotope ratios [74]. The ratios ^206^Pb/^207^Pb and ^208^Pb/^207^Pb were monitored for eight different primers and plotted against each other. The results demonstrate that for six out of eight primers it was possible to obtain a clear differentiation. The authors stated, however, that ICP-MS does not appear suitable for daily casework as it is a destructive technique [74].

#### 3.2.2. Laser Ablation- (LA-) ICP-MS

Although the analysis times using ICP-MS compared to SEM-EDX are relatively short, sample preparation requires time consuming digestion processes. Another drawback is the fact that ICP-MS is a bulk analysis technique and elemental information of discrete GSR particles cannot be obtained [75]. Therefore, studies were conducted to evaluate LA-ICP-MS for the detection of GSR particles. In LA, solid samples are vaporized by a short-pulsed high power laser for analysis. A gas stream (usually Ar) then transports the vaporized sample components into the ICP-MS enabling the determination of the elemental composition of GSR particles.

Abrego et al. studied the detection of seventeen isotopes (15 elements) considered to be present in particles either characteristic or consistent with GSR [76] from GSR tapes collected from the hands of shooters using four different weapons and ammunitions [75]. The flow rate of helium (carrier gas) and argon (nebulizer gas) was optimized to minimize elemental fractionation, which involves the nonstoichiometric ionization effects in the transient signal and is seen as one of the major drawbacks of LA-ICP-MS. Additionally, laser energy was optimized to obtain the best possible sensitivity. It was found that the ablation pattern “raster” was the most suitable one as it scanned the highest surface area and resulted in the highest number of particles detected. The detection of GSR particles was considered positive if isotopes of the three GSR components Sb, Ba, and Pb were detected. The results showed that LA-ICP-MS was able to detect GSR particles after only one shot was discharged. Higher amounts of GSR particles were found for pistols compared to revolvers. It should be mentioned that the other 12 elements of interest only gave low results. An advantage of LA-ICP-MS over SEM-EDX was found in the possibility for removal of epidermal cells from the GSR stubs using the laser. When GSR stubs are dabbed over the hands of a suspect, skin cells can also stick to the adhesive and overlay GSR particles. These particles are thus obscured for SEM-EDX analysis and can only be analysed if the skin cells are destroyed or new systems such as backscattered electron imaging (BEI) are connected to the SEM. This problem can be overcome by using LA-ICP-MS. The developed method showed good specificity and sensitivity; however it cannot give morphological information of GSR particles as offered by SEM-EDX [75]. Another important point to consider is that, in LA, the resolution is limited by the spot size diameter of the laser. In the study by Abrego et al. a laser was focused on the sample surface using the relatively large spot size diameter of 160 *μ*m [75]. This spot size diameter does not allow the resolution of adjacent small particles, for example, 5 *μ*m diameter, and therefore careful data interpretation is required to avoid incorrect conclusions. For example, adjacent particles containing individual heavy metal species may appear to be a single entity with the characteristics of GSR.

#### 3.2.3. ICP-MSF-MS or HR-ICP-MS

The combination of ICP and MSF-MS offering high mass resolution has also been applied to IGSR analysis.

Reis et al. [77] developed a sampling technique using swabs spiked with 2% ethylenediaminetetraacetic acid (EDTA) solution to collect IGSR from different areas of the hands of a shooter. EDTA forms metal complexes and is therefore thought to increase the extraction efficiency of swabbing for the GSR particles. Collection using EDTA was found to be superior to 2% diluted nitric acid or deionised water based on the recovery of Sb, Ba, and Pb from the hands of shooters before and after test-firings. The best recoveries were obtained from the collection of IGSR on areas near the thumb and forefinger. The concentrations of Pb and Sb after shooting a .38 calibre weapon (about 10 to 26 *μ*g/L) were found to be significantly higher than the concentrations before shooting (up to 0.17 *μ*g/L) and could be used as a tool for indicating the potential presence of GSR. The developed method including EDTA swabs and ICP coupled with an MSF-MS instrument achieved LODs of 0.045 *μ*g/L, 0.507 *μ*g/L, and 0.117 *μ*g/L for Sb, Ba, and Pb, respectively.

The developed collection and analytical method was further tested for the detection of IGSR from 9 mm and 0.40 in. calibre pistols [78]. The high sensitivity of HR-MSF-MS allowed the detection of GSR even from clean range ammunition, which usually leaves only a small quantity of IGSR. Ternary graphs displaying the relative percentages of the three components were constructed and allowed determination as to whether the data originated from a shooter or nonshooter. However, based on the concentrations of Pb, Ba, and Sb, it was not possible to distinguish between the two weapon types tested [78].

Freitas et al. examined the use of ICP-MSF-MS to study the possibility of differentiating weapons and ammunition based on the IGSR detected on different fabrics [79]. The results demonstrated that GSR does not persist on different fabrics reproducibly, as no significant differences in Sb, Ba, and Pb levels were found between the five tested fabrics, that is, microfiber, flannel, canvas, tergal (a polyester), and tricoline (a variety of cotton). Fabric blanks showed a significantly lower concentration of the three GSR characteristic compounds than fabric samples after shooting. Using ternary graphs, the authors could clearly distinguish fabrics before and after shooting and a characteristic pattern of distribution for pistols and round-barrel revolvers was determined [79].

A number of aspects of ICP-MS have contributed to the increasing interest in the application of this technique to IGSR analysis. The high sensitivity offered by ICP-MS is superior to that offered by SEM-EDX for inorganic GSR, for example, Sb, Ba, and Pb. Limits of detection in spiked swabs were in the low nanogram range, which is lower than concentrations expected in GSR samples (40–500 ng) [69] enabling all GSR samples tested by ICP-MS to be easily distinguished from the blanks. It can also be applied to the bulk analysis of gunshot wounds at any stage of decomposition and is superior to SEM-EDX analysis which is limited by the challenged sample collection at later stages of decomposition. In addition, the ability to measure isotope ratios has shown potential to distinguish between different primer types. A great advantage of ICP-MS is the high sample throughput in comparison to SEM-EDX. However, the destructive nature and the challenge of obtaining morphological information for single GSR particles limit its application to GSR detection and currently SEM-EDX remains the method of choice in most circumstances.

## 4. General Discussion and Conclusions

Mass spectrometry has been increasingly investigated in the field of GSR analysis for application to both inorganic and organic GSR.

The most common collection technique applied to OGSR is swabbing during which there is the potential for simultaneous collection of interfering substances on skin or garments. Therefore, a chromatograph is commonly placed in front of the MS instrument to separate the compounds of interest from any interference still present after extraction. The use of tandem mass spectrometry offers outstanding sensitivity and selectivity and chromatographic separation may only be necessary when compounds with similar *m*/*z* ratio, that is, isomers, are to be analysed. The great advantages offered by tandem MS explain its increasing use in OGSR analysis and other fields. The most sensitive tandem MS technique is QqQ-MS/MS allowing the identification and detection of OGSR in the low nanogram to picogram range, which is sufficiently sensitive for real case samples.

The ionization of the broad range of compounds present in OGSR has proven challenging as different ionization techniques may be required to obtain optimal results for all compounds. Hard ionization techniques, that is, EI, are only used in conjunction with GC for SPME analysis of volatiles in GSR, assisting with the estimation of time since discharge [23, 32]. The introduction of dual ionization techniques allows for the alternate use of ESI and APCI in positive and negative modes and the simultaneous detection of a wide range of the compounds likely to be present in OGSR [25]. From a forensic perspective, this is beneficial as it can maximise the information obtained regarding a firearm-related event. Therefore, the application of a dual ionization technique is of significant value for forensic analysis. Currently, the use of a dual ionization source coupled to a QqQ mass analyzer operated in MS/MS mode offers the best performance in most circumstances for OGSR analysis.

Different techniques mainly based on ToF-SIMS and ICP-MS have been evaluated for their applicability to ISGR analysis. Although these techniques may offer advantages in certain casework situations, for example, the collection and analysis of IGSR from decomposing tissues, and may detect the metals of interest with higher sensitivity, the method of choice for IGSR analysis remains SEM-EDX. Reasons for this include the relatively simple sample preparation, the capability for retrieval of elemental and morphological information for single GSR particles, and the fact that it is a nondestructive technique. Therefore, it seems unlikely that MS techniques will replace SEM-EDX in any foreseeable future.

While discussion for OGSR and IGSR was presented separately above, it must be remembered that these are two aspects of the same informational content borne by the GSR trace. The ability to analyse both aspects, hence potentially indicating the simultaneous presence of OGSR and IGSR, would greatly strengthen the value of the results. Finally, it is worth pointing out that any technique is only as good as it answers the questions being asked. Continuing research is required to realise the full potential of advanced analytical methods such as those presented here for application in the forensic sciences.

## Figures and Tables

**Table 1:** Analytical techniques applied to GSR and their abbreviations in alphabetical order.

Abbreviation	Instrumentation/technique
APCI	Atmospheric pressure chemical ionization
APPI	Atmospheric pressure photoionization
CE	Capillary electrophoresis
CI	Chemical ionization
DESI	Desorption electrospray ionization
EDX	Energy-dispersive X-ray detection
EI	Electron impact
ESI	Electrospray ionization
GC	Gas chromatography
HPLC	High performance liquid chromatography
ICP-MS	Inductively coupled plasma-mass spectrometry
IMS	Ion mobility spectrometry
LC	Liquid chromatography
LA	Laser ablation
LEI	Laser electrospray ionization
MS	Mass spectrometry or mass spectrometer
MS/MS	Tandem mass spectrometry
MSF	Magnetic sector field
Q	Quadrupole
QIT	Quadrupole ion-trap
QqQ	Triple quadrupole
QToF	Quadrupole-time of flight
SEM	Scanning electron microscopy
SIMS	Secondary ion mass spectrometry
SPE	Solid-phase extraction
SPME	Solid-phase microextraction
ToF	Time of flight
UHPLC	Ultrahigh performance liquid chromatography

**Table 2:** List of common organic explosives and additives used in the manufacture of propellant powders and primers [13, 22, 27, 28].

Compound	Abbreviation	Function
Nitroglycerin	NG	Explosive
1,2-Dinitroglycerin	1,2-DNG	Explosive
1,3-Dinitroglycerin	1,3-DNG	Explosive
Nitroguanidine	NGU	Flash suppressor
2,4,6-Trinitrotoluene	TNT	Explosive, sensitizer
2,3-Dinitrotoluene	2,3-DNT	Flash suppressor
2,4-Dinitrotoluene	2,4-DNT	Flash suppressor
2,6-Dinitrotoluene	2,6-DNT	Flash suppressor
3,4-Dintirotoluene	3,4-DNT	Flash suppressor
2-Amino-4,6-dinitrotoluene	2-A-4,6-DNT	Flash suppressor
4-Amino-2,6-dinitrotoluene	4-A-2,6-DNT	Flash suppressor
2-Nitrotoluene	2-NT	Explosive, flash suppressor
3-Nitrotoluene	3-NT	Explosive, flash suppressor
4-Nitrotoluene	4-NT	Explosive, flash suppressor
1,3,5-Trinitrobenzene	TNB	Explosive
1,3-Dinitrobenzene	1,3-DNB	Explosive
Nitrobenzene	NB	Explosive
Methyl centralite	MC	Stabilizer, plasticizer
Ethyl centralite	EC	Stabilizer, plasticizer
Butyl centralite	BC	Stabilizer, plasticizer
1,3-Benzenediol	Resorcinol	Stabilizer, plasticizer
Dimethyl phthalate	DMP	Plasticizer
N, N′-Diphenyl urea (Akardite I)	AKI	Stabilizer
N′-Methyl-N, N-diphenyl urea (Akardite II)	AKII	Stabilizer
N′-Ethyl-N, N-diphenyl urea (Akardite III)	AKIII	Stabilizer
Cresol	—	Stabilizer
Dinitro-*ortho*-cresol	DNOC	Stabilizer
Methyl phthalate	MP	Plasticizer
Ethyl phthalate	EP	Plasticizer
Diethyl phthalate	DEP	Plasticizer
Dibutyl phthalate	DBP	Plasticizer
Diamyl phthalate	DAP	Plasticizer
Camphor	—	Plasticizer
Diphenylamine	DPA	Stabilizer
2-Nitrodiphenylamine	2-NDPA	Stabilizer (derivative of DPA)
4-Nitrodiphenylamine	4-NDPA	Stabilizer (derivative of DPA)
4-Nitrosodiphenylamine	4-nDPA	Stabilizer (derivative of DPA)
N-Nitrosodiphenylamine	N-nDPA	Stabilizer (derivative of DPA)
2,4-Dinitrodiphenylamine	2,4-DNDPA	Stabilizer (derivative of DPA)
4,4′-Dinitrodiphenylamine	4,4′-DNDPA	Stabilizer (derivative of DPA)
Ethylene glycol dinitrate	EGDN	Explosive
Diethylene glycol dinitrate	DEGN	Sensitizer, plasticizer
Pentaerythritol tetranitrate	PETN	Explosive, sensitizer
Octahydro-1,3,5,7-tetranitro-1,3,5,7-tetrazocine	HMX	Explosive
Hexahydro-1,3,5-trinitro-1,3,5-triazine	RDX	Explosive
2,4,6-Trinitrophenylmethylnitramine	tetryl	Sensitizer (primer)
1,3-Diacetyloxypropan-2-yl acetate	Triacetin	Flash suppressor, plasticizer
Diazodinitrophenol	(DDNP)	Initiating explosive (primer)

**Table 3:** Summary of MS techniques applied to the analysis of different organic compounds found in GSR.

OGSR compounds	MS mode	Mass analyzer	Ionization technique	Mode	Scanning mode	LOD	Reference
DPA, NG, 2,4-DNT, EC, DBP	MS	MSF	CI	Positive	Full scan	20 pg (DPA)	[23, 38]

RDX, tetryl, TNT, PETN	MS	MSF	CI	Negative	Full scan	100 ng injected (TNT)	[39]

NG, DEGN, TNT, tetryl, 2,4-DNT, RDX, PETN, AN	MS	MSF	CI	Positive	Full scan	1–10 *μ*g injected	[40]

MC, EC, DPA,	MS	QToF	DESI	Positive	SIM	—	[58]

MC	MS/MS	QqQ	ESI	Positive	MRM	60 pg injected	[29]

MC, NG	MS/MS	QqQ	ESI	Positive, negative	MRM	—	[31]

DPA, 4-NO-DPA, 4-NO_2_-DPA, N-NO-DPA, 2,4-DNO_2_-DPA	MS/MS	QqQ	ESI	Positive	MRM	1.0 ng/mL (DPA), 0.5 ng/mL (N-NO-DPA), 2.5 ng/mL (4-NO_2_-DPA) (20 *μ*L injectetd)	[45]

DPA, 4-NO_2_-DPA, N-NO-DPA, 2,NO_2_-DPA, AK II, MC, EC	MS/MS	QqQ	ESI	Positive	MRM	5 *μ*g (EC), 6 *μ*g (MC), 20 *μ*g (2-NO_2_-DPA), 27 *μ*g (N-NO-DPA), 32 *μ*g (4-NO_2_-DPA), 34 *μ*g (DPA), 115 *μ*g (AK II)	[28]

TNT, RDX, PETN, NG, DPA, EC	MS/MS	QqQ	ESI	Positive, negative	MRM	300 pg (NG), 200 pg (TNT), 10 pg (RDX), 3 pg (PETN), 8 ng (DPA), 1 pg (EC)	[44]

4,4′-DNO_2_-DPA, N-NO-DPA, MC, 4-NO_2_-DPA, DPA, 2-NO_2_-DPA, EC, DBP, MC, DMP	MS	QIT	ESI	Positive	Full scan	—	[41]

EGDN, NG, TNT, PETN, RDX, HMX	MS	QIT	ESI	Negative	Full scan	—	[42]

MC, EC	MS/MS	QIT	DESI	Positive	MRM	5–60 pg/cm^3^ (MC), 6–70 mg/cm^3 ^(EC)	[30]

DPA, EC, 2,4-DNT, DEP, DBP, 2-NO_2_-DPA, 4-NO_2_-DPA, 2,3-DNT, 2,5-DNT, 2,6-DNT, TNT, MC, NT	MS	QIT	EI	Positive, negative	Full scan	—	[36]

HMX, RDX, tetryl, TNT, 2,4-DNT, NG, EGDN, PETN	MS	Q, QIT	ESI (Q), CI (QIT)	Negative	Full scan	—	[43]

NG, PETN, RDX, HMX, tetryl, TNT, 2,4-DNT, TNB, 1,3-DNB	MS	QIT	APCI	Negative	Full scan	1.06 ng/*μ*L (NG), 0.36 ng/*μ*L (PETN), 0.22 ng/*μ*L (RDX), 0.36 ng/*μ*L (HMX), 0.18 ng/*μ*L (tetryl), 0.04 ng/*μ*L (TNT), 0.07 ng/*μ*L (2,4-DNT), 0.19 ng/*μ*L (TNB), 0.29 ng/*μ*L (1,3-DNB) (10 *μ*L injected)	[47]

RDX, PETN, HMX, 1,2-DNB, tetryl, 3,4-DNT, 2,3-DNT, 2,6-DNT, 2,6-diamino-DNT, 4-amino-2,6-DNT, 1,3-DNB, 1,4-DNB, 2,5-DNT, 2,4-DNT, TNT, 3,5-DNT, 2-amino-4,6-DNT	MS	QIT	APCI	Positive, negative	Full scan	3.2 ng (RDX), 41.2 ng (PETN), 0.7 ng (HMX), 11.1 ng (1,2-DNB), 12.4 ng (2,4-diamino-6-NT), 5.6 ng (tetryl), 10.3 ng (3,4-DNT), 15.2 ng (2,3-DNT), 7.3 ng (2,6-DNT), 37.3 ng (2,6-diamino-4-NT), 39.0 ng (4-amino-2,6-DNT), 32.8 ng (1,3-DNB), 17.7 ng (1,4-DNB), 3.7 ng (2,5-DNT), 34.7 ng (2,4-DNT), 4.0 ng (TNT), 7.2 ng (3,5-DNT), 7.3 ng (2-amino-4,6-DNT), 11.4 ng (TNB)	[49]

TNT, 2,3-DNT, 2,4-DNT, 2,5-DNT, 2,6-DNT, 3,5-DNT, 1,2-DNB, 1,3-DNB, 1,4-DNB, TNB, 2-A-4,6-DNT, 4-A-2,6-DNT, 2,4-DA-6-NT, 2,6-DA-4-NT, RDX, HMX, tetryl, PETN	MS	QIT	APCI	Negative	Full scan	17 pg/mL (TNT), 8.9 pg/mL (2,3-DNT), 2.5 pg/mL (2,4-DNT), 8.8 pg/mL (2,5-DNT), 8.8 pg/mL (2,6-DNT), 24 pg/mL (3,5-DNT), 6.3 pg/mL (1,2-DNB), 28 pg/mL (TNB), 53 pg/mL (2-A-2,4-DNT), 34 pg/mL (4-A-2,6-DNT), 33 pg/mL (2,4-DA-6-NT), 2.5 pg/mL (2,6-DA-4-NT), 4.0 pg/mL (RDX), 8.8 pg/mL (HMX), 70 pg/mL (tetryl), 563 pg/mL (PETN) (80 mL injected)	[53]

DPA, 4-NO-DPA, N-NO-DPA, 2-NO_2_-DPA, 4-NO_2_DPA, 2,4′-DNO_2_-DPA, 4,4′-DNO_2_-DPA, DBP, DEP, DMP, EC, MC, NG, 2-NT, 3-NT, 4-NT, 2,3-DNT, 2,4′-DNT, 2,6-DNT, 3,4-DNT,	MS/MS	QqQ	ESI, APCI	Positive, negative	MRM	2.9 ng (DMP), 5.3 ng (2,4-DNT), 6.9 ng (2,6-DNT), 2.4 ng (2,3-DNT), 0.4 ng (3,4-DNT), 8.1 ng (2-NT), 6.9 ng (4-NT), 64 ng (NG), 9.1 ng (3-NT), 3.9 ng (4-NO-DPA), 9.6 ng (DEP), 1.5 ng (MC), 17 ng (4,4′-DNO_2_-DPA), 4.8 ng (4-NO_2_-DPA), 4.9 ng (N-NO-DPA), 12 ng (2,4′-DNO_2_-DPA), 13 ng (DPA), 7.4 ng (EC), 6.7 ng (2-NO_2_-DPA), 5.2 ng (DBP)	[25]

DPA, DMP, DBP, EC, MC, 4-NO_2_-DPA, N-NO-DPA	MS	TOF	LEI	Positive	Full scan	—	[54]

DBP, dioctyl sulfosuccinate (sodium salt), PDMS, EC, NC, NG	MS	TOF	SIMS	Positive	Full scan	—	[55]
